# Rod and cone photoreceptor cells produce ROS in response to stress in a live retinal explant system

**Published:** 2010-02-23

**Authors:** Lavinia Bhatt, Gillian Groeger, Kieran McDermott, Thomas G. Cotter

**Affiliations:** 1 Cell Development and Disease Laboratory, Biochemistry Department, Biosciences Institute, University College Cork, Cork, Ireland; 2 Department of Anatomy, Biosciences Institute, University College Cork, Cork, Ireland

## Abstract

**Purpose:**

The production of reactive oxygen species (ROS) can lead to oxidative stress, which is a strong contributory factor to many ocular diseases. In this study, the removal of trophic factors is used as a model system to investigate the effects of stress in the retina. The aims were to determine if both rod and cone photoreceptor cells produce ROS when they are deprived of trophic factor support and to demonstrate if the nicotinamide adenine dinucleotide phosphate (NADPH) oxidase (Nox) enzymes are responsible for this ROS production.

**Methods:**

Retinas were explanted from mice aged between postnatal days 8–10 and cultured overnight. The following morning, confocal microscopy combined with various fluorescent probes was used to detect the production of ROS. Each time peanut agglutinin (PNA), a cone photoreceptor marker, was used to facilitate orientation of the retina. Dihydroethidium and dihydrorhodamine 123 (DHR123) were used to determine which cells produce ROS. Subsequently, western blots of retinal serial sections were used to detect the presence of Noxs in the different retinal layers. The Nox inhibitor apocynin was then tested to determine if it altered the production of ROS within these cells.

**Results:**

Live retinal explants, viewed at high magnifications using confocal microscopy, displayed an increase in the fluorescent products of dihydroethidium and DHR123 upon serum removal when compared to controls. DHR123 fluorescence, once oxidized, localized to mitochondria and was found in the same focal plane as the PNA staining. This showed that cones and rods produced ROS when stressed. Retinal serial sectioning established that the photoreceptor layer expressed Nox4, dual oxidase (Duox) 1, and Duox2 at varying levels. Finally, the Nox inhibitor apocynin decreased the burst stimulated by the stress of serum removal.

**Conclusions:**

Confocal microscopy and PNA staining allowed differentiation of cell types within the outermost layers of the retina, demonstrating that both rods and cones generated ROS in response to the stress of serum deprivation. Nox4 was the most abundantly expressed Nox in the photoreceptor layer, but Duox1 and Duox2 were also present at detectable levels, and as apocynin reduced the levels of ROS produced, this implied that these proteins may play some role in this production.

## Introduction

The retina is known to produce reactive oxygen species (ROS) through its high consumption of oxygen. It consists of three main nuclear layers comprised of seven major cell types. Rod and cone photoreceptors transduce light into electrical signals, which in turn are processed by amacrine, horizontal, bipolar and ganglion cells. Müller glial cells provide support for the neuronal cells, yet under normal physiologic conditions, these retinal cells possess many redox regulatory systems that control the normal production of ROS. This group of molecules, once considered as simple byproducts of oxygen consumption, are now recognized as important signaling molecules in their own right [1]. In certain disease conditions, the amount of ROS produced may become excessive, and so the retina undergoes oxidative stress, causing damage to the cells and eventual loss of vision. Increased oxidative stress is associated with many ocular diseases, such as retinitis pigmentosa [2] and age-related macular degeneration [3], and antioxidants have recently been shown to have therapeutic benefit for these and similar diseases [4-6]. Interestingly, it has also been recently demonstrated that low levels of ROS can stimulate a prosurvival response [7].

In recent years, the nicotinamide adenine dinucleotide phosphate (NADPH) oxidase (Nox) family of enzymes has become recognized as an important generator of ROS in many tissues as it controls many signaling pathways, such as cell migration, proliferation, survival, and death. Its original member, first called gp91^phox^ and now termed Nox2, was shown to generate high amounts of superoxide in phagocytic cells to kill pathogens. Over the past 10 years, there have been six other members of the family discovered—Nox1, Nox3–5, and dual oxidase (Duox) 1 and 2, which are expressed in many different cell types (for review see [8]). All Nox enzymes are known to generate ROS through the transfer of electrons from NADPH via intermediates to oxygen. Nox proteins have been shown to be important in several disease conditions, ranging from various cancers [9-11] to neuronal diseases [12,13].

The presence of Noxs in the retina is a new area of research with few publications to date. Given the critical role of ROS in the retina and in retinal diseases, specific knowledge of the role of Nox proteins could aid in the design of future therapies. We recently demonstrated that some members of the Nox family are expressed in the retina [14], while Usui et al. [2] showed that Noxs in general contribute to ROS production and hence degeneration of the retina in a model of retinitis pigmentosa. It still remains to be determined if both cone and rod photoreceptor cells in the retina produce ROS when the retina is stressed and if one Nox member in particular is responsible for this production.

A key obstacle to overcome to achieve our aims is the measurement of ROS production in real time in the retina. In the past this has proved challenging, with previously published methods relying on staining in fixed tissue [2] or flow cytometry work on trypsinized retinal cells [15] or detection of indirect products of oxidative damage, such as lipid hydroperoxide [16] and 4-hydroxy-2-nonenal protein adducts [17]. We recently made a small advancement in this area by using an inverted microscope with time-lapse capabilities to show how quickly ROS can be produced [14], but this monitored the retina globally and no differentiation between retinal layers or cells was possible. Here, a method is used that relies on the use of confocal microscopy, with specific markers to determine which cells in the photoreceptor layer generate ROS.

The first aim of the present study was to determine if both rod and cone photoreceptor cells produce ROS when the retina is challenged by a stress, namely serum removal. Confocal microscopy combined with a cone marker and ROS indicators demonstrated that both cones and rods generate ROS in these circumstances. The second aim was to examine which, if any, of the Nox proteins is responsible for this ROS production. This was achieved using western blotting of serial sections of retinal layers to reveal the Nox expression pattern throughout all the layers of the retina. Finally, a Nox inhibitor was used to demonstrate that it could reduce the ROS produced in the photoreceptor cells.

## Methods

### Reagents and antibodies

Peanut agglutinin (PNA) conjugated to fluorescein or rhodamine was purchased from Vector Laboratories (Peterborough, UK). The superoxide indicator dihydroethidium (DHE) and general ROS indicator dihydrorhodamine 123 (DHR123) were purchased from Molecular Probes (Leiden, the Netherlands). The antibodies used were anti-gp91phox (Nox2; BD Transduction Laboratories, Oxford, UK), anti-Nox4 and anti-Duox2 (Abcam, Cambridge, UK), anti-rhodopsin (Thermo Fisher Scientific, Cheshire, UK), anti-Duox1 and anti-Thymocyte differentiation antigen 1 (Thy-1; Santa Cruz Biotechnology, Santa Cruz, CA), anti-ceh-10 homeodomain containing homolog (CHX10; Millipore, Cork, Ireland), and anti-tubulin (Sigma-Aldrich, Dublin, Ireland). The secondary peroxidase-conjugated antibodies were purchased from Dako (Glostrup, Denmark). Apocynin was obtained from Calbiochem (Nottingham, UK).

### Retinal preparations

All experiments were conducted in accordance with the ARVO statement for use of Animals in Ophthalmic and Vision Research and approved by the University College Cork Animal Ethics Committee. C57/BL6 mice were obtained from Biologic Service Unit (University College, Cork, Ireland). Mice were decapitated (below the age of p11, decapitated without any anesthetic) between postnatal days 8 and 10, and their eyes enucleated. Forty mice were used in these experiments. The lens, anterior segment, vitreous body, retinal pigment epithelium, and sclera were removed before the retina was mounted flat with photoreceptor side down, either in a glass-bottom dish (35 mm Petri dishes with 14 mm microwells and coverslip thickness of 0.16–0.19 mm; MatTek Corporation, Ashland, MA) for confocal imaging or on top of a nitrocellulose insert (Millipore, Billerica, MA) in six-well plates (Sarstedt AG & Co, Wexford, Ireland) for the experiments on postfixed retinas. All explants were cultured in R16 medium (recipe from Dr. PA Ekstrom, Wallenberg, Retina Center, Lund University, Lund, Sweden, ingredients are listed in Table 1) and left to recover overnight.

**Table 1:** The individual components used to make up the medium in which retinal explants were cultured.

**Chemical**	**Supplier**	**Concentration**
R16 powder	Gibco/Invitrogen	(as supplied)
NaHCO_3_	Sigma-Aldrich	(2.8 g in 1 l)
NaSeO_3_	Sigma Aldrich	7.9 μg/ml
MnCl_2_	Sigma Aldrich	1 μg/ml
CuSO_4_	Sigma Aldrich	2.5 μg/ml
Biotin	Sigma Aldrich	0.1 μg/ml
Ethanolamine	Sigma Aldrich	1 μg/ml
BSA	Sigma Aldrich	0.002%
Corticosterone	Sigma Aldrich	0.02 μg/ml
L-cysteine	Sigma Aldrich	7.09 μg/ml
DL-α-tocopherol	Sigma Aldrich	1 μg/ml
DL-α-tocop acetate	Sigma Aldrich	1 μg/ml
Linoleic acid	Sigma Aldrich	1 μg/ml
Linolenic acid	Sigma Aldrich	1 μg/ml
3,3′ 5-Triiodo L thyronine sodium	Sigma Aldrich	0.002 μg/ml
Thiamine HCl	Sigma Aldrich	2.77 μg/ml
Thiocitic Acid	Sigma Aldrich	0.045 μg/ml
Transferrin	Sigma Aldrich	10 μg/ml
Vitamin B12	Sigma Aldrich	0.31 μg/ml
Glutamine	Sigma Aldrich	25 μg/ml
Vitamin C	Sigma Aldrich	100 μg/ml
Glutathione	Sigma Aldrich	1 μg/ml
Insulin	Sigma Aldrich	0.02 μg/ml
Progesterone	Sigma Aldrich	0.0063 μg/ml
Pyruvate	Sigma Aldrich	50 μg/ml
Retinol	Sigma Aldrich	0.1 μg/ml
Retinyl acetate	Sigma Aldrich	0.1 μg/ml

### Peanut agglutinin staining in postfixed tissue

In certain preliminary experiments, retinas were dissected, explanted onto a nitrocellulose insert, quickly washed in PBS (137 mM NaCl, 2.7 mM KCl, 10 mM Na_2_HPO_4_, and 2 mM KH_2_PO_4_; pH=7.4), Shandon cryochrome (Thermo Fisher Scientific, Cheshire, UK) added on top of the insert and covering the retina, and frozen at −80 °C. Subsequently, 7 μm sections were cut on a cryostat (Leica CM1950; Leica, Wetzlar, Germany) and postfixed in 10% formalin for 20 min at room temperature (RT; 20–22 °C). These sections were rinsed in PBS, followed by incubation with PBS containing 0.1% Tween-20 (PBS-Tween; Sigma-Aldrich) for 30 min at RT. Subsequently, the sections were incubated with 50 μg/ml rhodamine-conjugated PNA and 1 μg/ml Hoechst 33342 (Sigma-Aldrich) in PBS-Tween for 1 h at RT, rinsed three times in PBS-Tween for 10 min each time, and given one last rinse in PBS. All postfixed sections were mounted and viewed under a fluorescence microscope (Leica DM LB2; Leica) using the appropriate filters.

### Reactive oxygen species production using confocal laser scanning microscopy

Retinal explants used for live confocal imaging were pre-incubated for 1 h at 37 °C with either fluorescein conjugated to PNA (10 μg/ml) and 10 μM DHE, or rhodamine conjugated to PNA (10 μg/ml) and 10 μM DHR123. After this incubation with the probes, the explants were rinsed in PBS once and incubated with basal medium (BM), which was medium without the addition of any growth factors, or full medium (FM) as stated. Where indicated, retinal explants were treated with 4 mM apocynin for 2 h before the addition of PNA+DHE or PNA+DHR123, at the concentrations described above. This concentration of apocynin was maintained with the dyes in the PBS wash and in the medium surrounding the tissue for imaging.

Confocal microscopy was performed at 37 °C. Live images of the retinal explants were acquired by an Olympus Fluoview1000 inverted confocal laser scanning microscope facilitated with multiline argon lasers 458, 488, and 515 nm, helium/neon laser red 633 nm, helium/neon laser green 543 nm, and the UV laser diode laser lines (Mason Technology, Dublin, Ireland). Retinal explants were examined and imaged with a UPlanSApo 60× oil immersion objective (1.35 numerical aperture; Olympus Optical Gmbh, Hamburg, Germany). Retinal explants stained with fluorescein conjugated to PNA or DHR123 dyes were excited at 488 nm, and emissions were collected at 530 nm. When either rhodamine conjugated to PNA or the ROS detector DHE was used, the dyes were excited at 543 nm, while emissions were collected at 590 nm. Additionally, retinal explants stained with Hoechst were excited at 358 nm, with emissions collected at 461 nm.

The laser power and detection settings were kept constant in each set of treatments to allow direct comparison of retinal explants treated with BM or FM with or without apocynin. Emissions were collected by sequential scans between two channels of interest to avoid artifacts or bleed through between the two channels. Images were acquired at a 1024×1024-pixel resolution in the frame-scan mode. These settings were maintained at all times when imaging. Individual confocal slices acquired were either 1.6 μm or 2 μm thick, and a total of 10–25 slices were collected. In some cases, representative slices have been shown in the figures, as indicated in the figure legends.

Images were acquired, stored, and visualized with Olympus Fluoview 1000 software (Mason Technology). Images were analyzed by constructing montages using the Image J 1.42v software and were labeled in Adobe Illustrator 9.0 (Adobe systems; Dublin, Ireland).

### Western blotting of serial sections of the retina

This method was performed as previously described [18,19]. Briefly, a mouse eye was enucleated and retinal dissection performed, as described above, and flattened onto a nitrocellulose insert, with the ganglion cells uppermost. The retina still attached to the nitrocellulose was then flat mounted on a glass coverslip. Once frozen, it was aligned with the cutting surface of the cryostat and 30 μm serial sections were cut, dissolved in 15 μl of sodium dodecyl sulfate-polyacrylamide gel electrophoresis (PAGE) sample buffer, and subjected to western blotting, using standard techniques as previously described [14]. Samples were separated on 10% sodium dodecyl sulfate-polyacrylamide gels, and transferred to nitrocellulose membranes, which were subsequently blocked with 5% non-fat dry milk in Tris-buffered saline with 0.1% Tween-20 for 1 h at room temperature. Following overnight incubation at 4 °C with the primary antibody at 1:1000 dilution (apart from the following: anti-Duox2, 1:500; anti-tubulin at 1:5000), membranes were washed and incubated with peroxidase-linked secondary antibodies, and the signal detected using enhanced chemiluminescence substrate (ThermoScientific, Northumberland, UK).

### Image analysis

All experiments were repeated at least three times independently, and any images shown are representative of the results obtained. When any adjustment was made to the contrast of images post collection, this was done for the entire image. Within experiments, adjustments were kept constant between the different treatments to allow direct comparisons of the fluorescence levels.

## Results

### Confocal microscopy on live explants stained with peanut agglutinin allows differentiation between rod and cone photoreceptor cells

**Figure 1:**
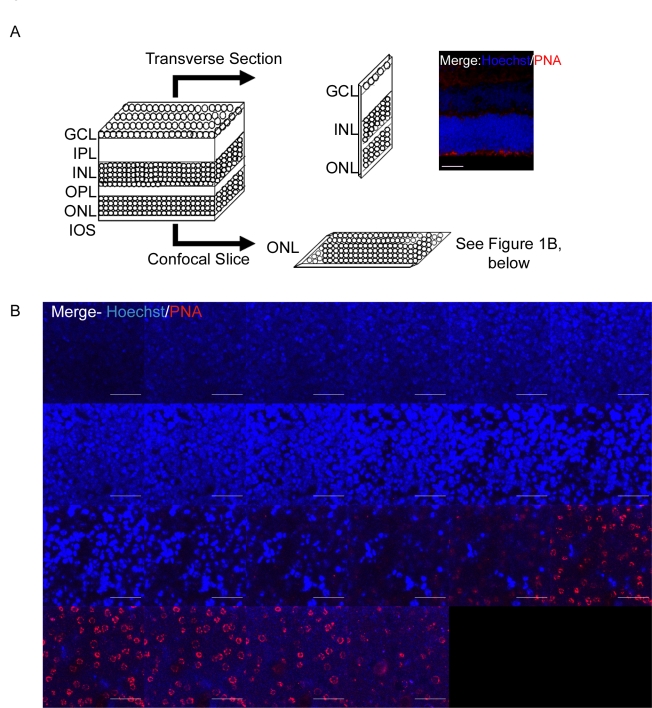
Confocal microscopy in conjunction with peanut agglutinin (PNA) staining allows differentiation between rods and cones in live explants. **A**: A schematic representation of the retina and its layers (ganglion cell layer [GCL], inner plexiform layer [IPL], inner nuclear layer [INL], outer plexiform layer [OPL], outer nuclear layer [ONL], inner and outer segments [IOS]) to illustrate the difference between a transverse section and a confocal slice. The image is a transverse section stained with rhodamine-PNA and Hoechst to allow comparison with the confocal images of **B**. The scale bar represents 25 μm. **B**: Hoechst/PNA staining of the ONL on a whole-mount retinal explant by confocal microscopy. Explants were cultured photoreceptor side facing down. Retinal whole mounts were stained with Hoechst/PNA for 1 h at 37 °C before live imaging by an inverted confocal microscope. PNA, the cone-specific marker, was used to bring the photoreceptor layer into focus. Slices preceding the PNA-stained layers are the Hoechst-positive nuclei in the ONL. These data are typical of at least three different experiments. Confocal slices collected were 1.6 μm thick. The scale bar represents 10 μm.

To establish if it would be possible to use confocal microscopy to distinguish between the outer and inner segments of photoreceptors and the outer nuclear layer (ONL), retinas were co-stained with Hoechst and PNA. When using confocal microscopy on retinal whole mounts, the retina is viewed at right angles to the more commonly used transverse sections, as indicated by the diagram in Figure 1A. This means that confocal slices are equivalent to thin longitudinal or serial sections through one layer of the retina at a time. The confocal microscope used in this instance was an inverted microscope, and as retinas were cultured photoreceptor side down, this allowed good access to the inner and outer segments and ONL (Figure 1A).

PNA stains specifically the cone plasma membrane around the cone inner and outer segments in the photoreceptor layer of the retina [20], and PNA positive cells are detected in mice from birth throughout development [21]. As expected, the PNA staining detected here did not co-localize with the nuclear stain Hoechst, implying that it was adjacent to but not within a nuclear layer (Figure 1B). Comparing this staining to a conventional transverse section also stained with PNA and Hoechst (Figure 1A), the two match well. These results gave us confidence that PNA staining could be used in conjunction with ROS indicators to determine if photoreceptors generate ROS in response to serum removal.

### Reactive oxygen species are generated in rods and cones of the photoreceptor layer

**Figure 2:**
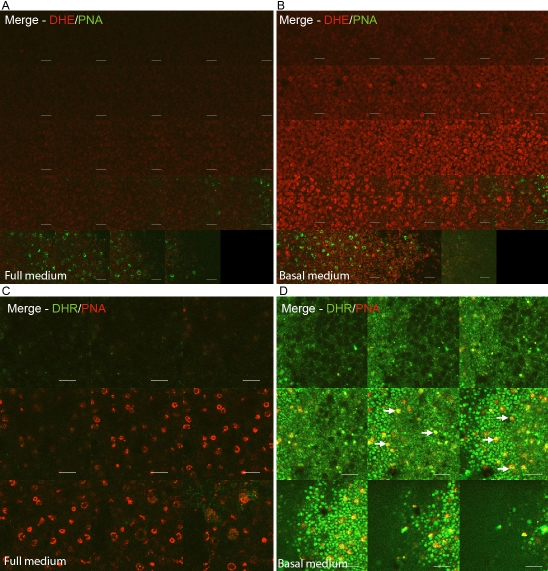
The reactive oxygen species (ROS) burst is generated in both rods and cones of the photoreceptor layer as determined by confocal microscopy. Prior to imaging, retinal whole mounts were treated with either dihydroethidium (DHE)/peanut agglutinin (PNA; **A**,** B**) or dihydrorhodamine (DHR)/PNA (**C**, **D**) for 60 min at 37 °C. Arrows point to the PNA-positive cones in red that produced ROS, as indicated by oxidized DHR123 staining in green (**D**). These data were typical of at least three independent experiments. Confocal slices collected were 1.6 μm thick. The scale bar represents 10 μm.

By confocal microscopy the photoreceptor layers of various explanted retinas were examined to determine if they generate ROS when stressed by serum removal. PNA was used in combination with either DHE or DHR123. DHE is converted into ethidium when it interacts with superoxide, and ethidium binds to DNA, fluorescing brightly. The ONL did not display any ethidium fluorescence when explants were treated with FM (Figure 2A). In contrast, when explants were treated with BM, a bright red, fluorescent, nuclear staining was detected in the ONL (Figure 2B).

DHR123 was the second ROS indicator used as it has different properties to DHE. DHR123 is an uncharged and nonfluorescent probe that diffuses passively across membranes. When DHR123 is oxidized, rhodamine123 (RH123) is generated, which displays an intense green fluorescence [22] and is known to accumulate in mitochondria [23]. Due to its green fluorescence, it was paired with rhodamine-conjugated PNA in these experiments. When retinal explants were treated with FM, the PNA-positive layer displayed a minimal amount of RH123 fluorescence (Figure 2C); when they were stressed by treatment with BM, there was bright green RH123 fluorescent staining (Figure 2D).

This RH123 staining localized in the same focal plane as the selectively stained PNA-positive cones. Co-localization between rhodamine-conjugated PNA and RH123 was demonstrated when red and green pixels overlapped, displaying a yellow merge (as indicated by arrows in Figure 2D). This degree of co-localization was somewhat unexpected as PNA is an extracellular marker, while mitochondria reside within cells, but it agrees with a recent paper from Bianchini et al. [24], which showed that mitochondrial markers also stain the extracellular membrane of photoreceptor outer segments. There were also some cells that showed the expected pattern of a bright ring of red surrounding an inner disc of green. Both of these patterns marked cones that produced ROS when stressed. Furthermore, it was observed that the RH123 fluorescence not only came from the PNA-positive cones but also from other cells that were on the same focal plane. The majority of these cells are likely to have been rods.

Together, these results fulfill our first aim as they demonstrate that both rods and cones increase their ROS production upon trophic factor deprivation (Figure 2). This level of differentiation was possible due to the use of confocal microscopy with a marker for cones and general ROS probes.

### Nox family members display differential expression within retinal layers

**Figure 3:**
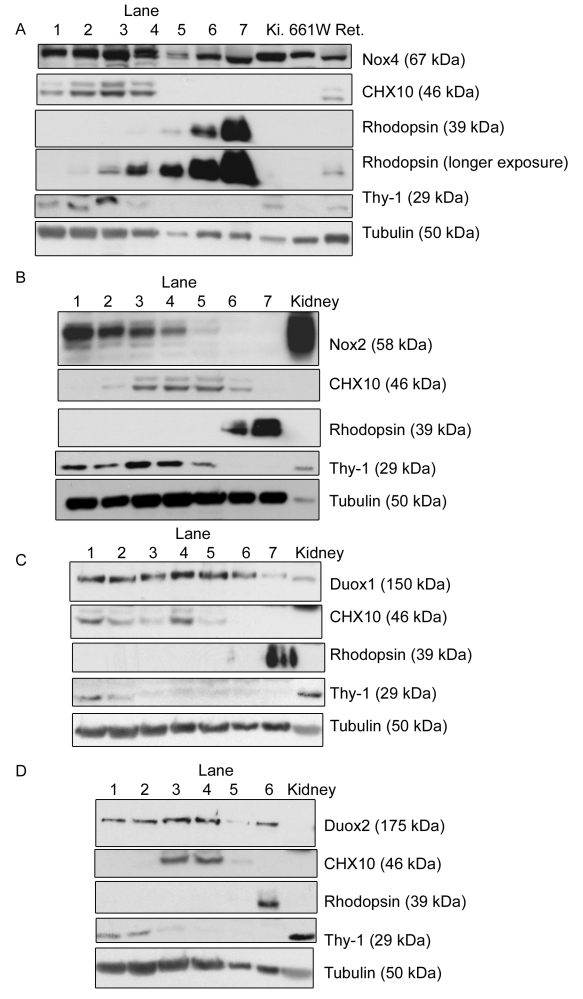
The expression pattern of nicotinamide adenine dinucleotide phosphate oxidase (Nox) 4, Nox2, dual oxidase (Duox) 1, and Duox2 in retinal layers is demonstrated through western blotting of serial sections of the retina. In all parts, Lane 1 represents the first section removed from the ganglion cell side of the retina, and lane 6/7 is the final section obtained from that retina and represents the outermost layer of the photoreceptor cell side of the retina. Thymocyte differentiation antigen 1 (Thy-1) served as a marker of ganglion cells, rhodopsin of photoreceptor cells, and Ceh-10 homeodomain containing homolog (CHX10) of rod bipolar cells, illustrating that the technique functioned well to separate the retinal layers (middle panels of **A**, **B**, **C**, **D**). In part **A**, the three lanes on the far right of the gel are control lanes, which are marked as follows, kidney (K_i_.; which is known to be a high expressor of Nox4), 661W cells, and a whole retinal lysate (Ret.) to demonstrate the similarity between this and our previous paper [14]. In **B**-**D**, only the kidney control was maintained. The expression of Nox4 throughout all of the retinal layers is demonstrated (**A**). Nox2 was expressed in most of the retinal layers, with highest expression in the ganglion cell side and lowest at the photoreceptor side (**B**). Duox1 (**C**) and Duox2 (**D**) showed similar expression patterns, with their highest expression levels being in the middle lanes. Tubulin was used throughout as a loading control. Blots are representative of at least three independent experiments.

We have previously demonstrated that Noxs contribute to the generation of ROS in both the 661W photoreceptor-derived cell line [25] and in whole retinal explants [14], which have since been confirmed in an animal study [2]. To establish which of the retinal layers express which members of the Nox family, serial sectioning of flat-mounted retinas was used. In this technique, 30 μm sections of a retina were cut using a cryostat. The retina was aligned flat with the blade so the first section would contain mainly ganglion cells, and then the second would consist of the inner plexiform layer and so forth through the retinal layers (see Figure 1A for reference, with the blade positioned at the ganglion cell layer first). Blots were probed with a marker of ganglion cells (Thy-1), rod bipolar cells (CHX10), and photoreceptor cells (rhodopsin), which showed the expected differential expression pattern of these proteins, thus validating the technique (Figure 3A-D) pioneered by Sokolov et al. [19,26] and further modified in this laboratory [18,27,28]. All the Nox antibodies used in this study detected more than one band when used for western blotting, which meant that they were unsuitable for use in immunohistochemistry techniques.

Nox5 is not found in rodents [29], Nox3 is mainly associated with the inner ear [8], and the mouse retina does not express Nox1 at this age [14], so these members of the Nox family were not considered in this study. The expression patterns of the other four members, namely Nox2, Nox4, Duox1, and Duox2, were investigated. Nox4 was expressed at varying levels across all layers but with significant expression in the photoreceptor layer (Figure 3A). Kidney lysate was used as a positive control as it has high levels of Nox4 expression and is known to also express Nox2 [30]. Nox2 was mainly expressed in layers close to and coinciding with the ganglion cell layer, with minimal expression in the rhodopsin-positive layer (Figure 3B). Duox1 and Duox2 showed a similar pattern, but with the heaviest expression lying in between the rhodopsin-positive photoreceptor layers and the Thy-1-positive ganglion cells (Figure 3C,D). This led to the possibility that at least one of these Nox proteins was responsible for the ROS generation in the photoreceptor layer documented in the preceding section.

### Reactive oxygen species production blocked by apocynin

**Figure 4:**
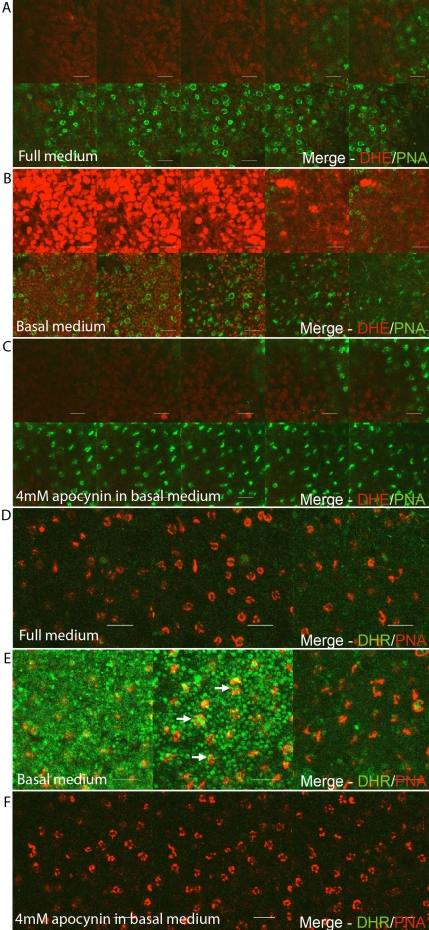
Apocynin effectively blocks the burst generated in the photoreceptor layer. Retinal explants were treated with either dihydroethidium (DHE)/peanut agglutinin (PNA; **A**, **B**, **C**) or dihydrorhodamine (DHR)/PNA (**D**, **E**, **F**). In some cases, explants were pretreated with 4 mM apocynin for 3 h at 37 °C (**C**, **F**). Before imaging, explants were treated with full medium (**A**, **D**), basal medium (**B**, **E**), or apocynin in basal medium (**C**, **F**). Retinal whole mounts were cultured and oriented as described in the Material and Methods. Arrows point to the PNA-stained cones (red) that produce oxidized DHR123 (green). These data were typical of at least three independent experiments. Confocal slices collected were 2.0 μm thick. Representative slices are shown. The scale bar represents 10 μm.

To further investigate if the Nox family contributed to the generation of ROS in this system, apocynin, a known Nox inhibitor [8] and antioxidant [31], was used (Figure 4). When retinal whole mounts were treated with 4 mM apocynin and BM, superoxide radicals (Figure 4C) and ROS (Figure 4F), otherwise generated by BM in the photoreceptor layer (Figure 4B,E), were substantially reduced to levels seen when they were treated with FM (Figure 4A,D). Therefore, this second set of results fulfills the second aim by demonstrating that Nox enzymes are the possible source of ROS production under these conditions.

## Discussion

In this paper, we focused on the outermost layers of the retina because a previous study [1] using the rd1 mouse model of retinitis pigmentosa demonstrated these cells were responsible for generating ROS in that system. The stress of serum removal was used as the model here to examine the effects of stress on photoreceptors within a living retinal explant. Confocal microscopy imaging of live explants enabled differentiation between the cells in this layer and revealed that ROS were generated from both PNA-positive cones and cells devoid of PNA staining, the majority of which would be rods (Figure 2D). Western blotting of serial retinal sections demonstrated that, in the photoreceptor layer, Nox4 was the most abundantly expressed Nox family member and that Duox1 and Duox2 were also detectable (Figure 3). This expression pattern was compatible with the fact that apocynin, a known Nox inhibitor, reduced ROS levels greatly.

This study advances previously published work by demonstrating that both rods and cones produce ROS when stressed. This was somewhat unexpected because in disease models, and in retinitis pigmentosa specifically, rods are known to die first, with cones dying secondarily [32]. There are several theories to explain why this should be the case, but none of them have been definitively proven. It is important to identify the precise cause(s) of both rod and cone cell death, as it is the secondary loss of cones that results in total blindness [33]. One theory that has led to the development of successful therapy is that the death of rods leads to higher oxidative stress in the retina, which eventually kills the cones. To prove this theory, Komeima et al. demonstrated that antioxidants promote cone survival and vision in rd1 mice as they age [32]. As we have demonstrated here that both rods and cones produce ROS in response to a stress stimulus (Figure 2), the question remains as to why rods would then die preferentially and only cones be saved when treated with antioxidants. It implies possibly that rods are more sensitive to ROS and so die first, which then probably increases the oxidative stress in the retina even further. By adding antioxidants, cones are helped in their struggle to survive any oxidative insult. It is also probable that the response of cells is stress specific, and if another model were used, such as hypoxia, it is likely that these retinal cells may not respond in the same way.

This study also demonstrates that the Nox family of enzymes probably plays a role in ROS production in these photoreceptor cells, as they are expressed in this layer. However, there is differential expression of Nox2, Nox4, Duox1, and Duox2 throughout the retina, implying that Noxs may have many varied roles in this tissue. This multiple expression of Noxs within one tissue is not uncommon as it has already been reported in adipose tissue [34,35] and various cardiac tissues, such as arteries (reviewed by Griendling [36]). There is some controversy over the Nox inhibitor apocynin as it can also act as an antioxidant [31], depending on several variables. The reason why we are confident that Nox enzymes play an important role in the ROS generation detailed here is because two other studies, independently of this one, have also shown that Nox inhibitors decrease ROS production in the retina. The first of these used a different Nox inhibitor (diphenyliodonium) [14] and retinal explants to show a global decrease in ROS production. The second used apocynin at a different concentration, but it was injected into animals and successfully decreased ROS levels in the retina [2]. This second study also tested another inhibitor of flavo-containing proteins (allopurinol), which had no effect on ROS production [2]. Taking these pieces of evidence together with the fact that there are four Nox proteins expressed throughout the retina, including in the photoreceptor layer (Figure 3), strongly implies that the Nox family generates ROS in this case.

Unlike “house-keeping” proteins, such as tubulin, which remain relatively constant throughout the retina, all four Nox proteins investigated were differentially expressed through the retinal layers. This varied expression could give rise to another strategy in the design of antioxidant therapies for ocular diseases as it could allow a more targeted approach of Nox4, the member that appears to be most highly expressed in the photoreceptors. As Nox2 and Nox4 have quite different protein structures, being only 39% homologous [37], and different regulatory mechanisms [37,38], the direct targeting of Nox4 within the retina to prevent this large increase in ROS may be possible, while allowing the other cells to maintain their normal ROS production through the activity of Nox2, Duox1, and Duox2. The fact that four of the seven Nox members are expressed in the retina shows how important this family, and hence ROS generation, is likely to be for the normal maintenance of the retina. It also highlights a potential pitfall in this area as it means that there is likely to be some form of redundancy in the function of these Noxs in the retina. So, if one specific member of the family was targeted by a therapy, it is likely that another one could replace its function.

We have demonstrated that both cones and rods produce ROS when stressed through the action of Nox enzymes. This has advanced the knowledge of the role of Nox proteins in the retina and provided a possible way to target ROS production in the photoreceptor layer over that in the other retinal layers. Using confocal microscopy in conjunction with PNA allowed a comprehensive analysis of ROS production in the ONL, including good differentiation between its cells. This detailed knowledge of ROS production in the retina should allow for further tailoring of therapies designed to combat ocular disease involving high levels of oxidative stress.
